# Retraction Note: m6A mRNA methylation initiated by METTL3 directly promotes YAP translation and increases YAP activity by regulating the MALAT1-miR-1914-3p-YAP axis to induce NSCLC drug resistance and metastasis

**DOI:** 10.1186/s13045-023-01416-6

**Published:** 2023-02-22

**Authors:** Dan Jin, Jiwei Guo, Yan Wu, Jing Du, Lijuan Yang, Xiaohong Wang, Weihua Di, Baoguang Hu, Jiajia An, Lingqun Kong, Lei Pan, Guoming Su

**Affiliations:** 1 Clinical Medical Laboratory, Binzhou Medical University Hospital, Binzhou, 256603 People’s Republic of China; 2 Cancer Research Institute, Binzhou Medical University Hospital, Binzhou, 256603 People’s Republic of China; 3 Department of Thyroid and Breast Surgery, Binzhou Medical University Hospital, Binzhou, 256603 People’s Republic of China; 4 Department of Pain, Binzhou Medical University Hospital, Binzhou, 256603 People’s Republic of China; 5 Department of Gastrointestinal Surgery, Binzhou Medical University Hospital, Binzhou, 256603 People’s Republic of China; 6 Department of Clinical Laboratory, Binzhou Medical University Hospital, Binzhou, 256603 People’s Republic of China; 7 Department of Hepatobiliary Surgery, Binzhou Medical University Hospital, Binzhou, 256603 People’s Republic of China; 8 Department of Respiratory and Critical Care Medicine, Binzhou Medical University Hospital, , Binzhou, 256603 People’s Republic of China; 9 Department of Nursing, Binzhou Polytechnic University, Binzhou, 256603 People’s Republic of China

**Retraction Note: Journal of Hematology & Oncology (2019) 12:135** https://doi.org/10.1186/s13045-019-0830-6

The Editor in Chief has retracted this article because of a number of further image irregularities in the figures were found after publication of a correction [1]. Specifically, there were a number of concerns related to the images and sizes of the tumours in Fig. 6c, d and i, as well as the partial overlaps in Fig. 6e and the corrected Fig. 6k. Additionally, the concerns related to potential repeated elements in the Western blots in Figs. 1a, 2a and 4e have remained unaddressed. Therefore, the Editor has lost confidence in the integrity of the article's findings.

Jiwei Guo does not agree to this retraction. Dan Jin, Yan Wu, Jing Du, Lijuan Yang, Xiaohong Wang, Weihua Di, Baoguang Hu, Jiajia An, Lingqun Kong, Lei Pan and Guoming Su did not respond to correspondence from the Editor about this retraction.
